# Estimation of Antioxidant Consumption in an Adolescent Population from a School in Pachuca de Soto, Mexico: A Cross-Sectional Study by Convenience Sample

**DOI:** 10.3390/pediatric18010023

**Published:** 2026-02-04

**Authors:** Eli Mireya Sandoval-Gallegos, Alejandra López-García, Karen Rubí Escamilla-Gutiérrez, José Arias-Rico, Quinatzin Yadira Zafra-Rojas, Esther Ramírez-Moreno, Araceli Monter-Arciniega, Nelly del Socorro Cruz-Cansino, Alma Delia Román-Gutiérrez, Zacnicté Olguín-Hernández

**Affiliations:** 1 Academic Area of Nutrition, Institute of Health Sciences, Autonomous University of the State of Hidalgo, Pachuca 42130, Mexico; eli_sandoval7987@uaeh.edu.mx (E.M.S.-G.); esther_ramirez@uaeh.edu.mx (E.R.-M.); mo270469@uaeh.edu.mx (A.M.-A.); 2 Academic Area of Nursing, Institute of Health Sciences, Autonomous University of the State of Hidalgo, Pachuca 42130, Mexico; 3 Academic Area of Chemistry, Institute of Basic Sciences and Engineering, Autonomous University of the State of Hidalgo, Tulancingo-Pachuca Highway Km 4.5, Carboneras Neighborhood, Mineral de la Reforma 42184, Mexico

**Keywords:** phenolic compounds, antioxidant, adolescents

## Abstract

Background: Adolescence is a critical stage for establishing lifelong dietary habits and preventing non-communicable diseases through adequate intake of bioactive compounds. Numerous studies have thoroughly examined the antioxidant profile of traditional diets such as the Mediterranean diet. In contrast, current research provides limited insights into the antioxidant properties of foods typically consumed by Mexican adolescents. Objective: So, this study aimed to quantify the total phenolic compound (TPC) content and antioxidant capacity (AC) of frequently consumed foods and to estimate dietary intake in Mexican adolescents. Methodology: The selected food groups were identified based on their frequency of consumption by 15% or more of the adolescent population, considering those that have demonstrated a sufficient quantity of antioxidants. It was analyzed TPC and ABTS•^+^ and DPPH• to determine the antiradical activity of the analyzed samples. Results: The estimated daily intake of TPC was 1484.01 mg GAE/person, while AC intake was 345.67 mg AAE/person (ABTS•^+^) and 5399.14 µmol TE/person (DPPH•). Cereals and fruits were the major contributors to total antioxidant intake, while the contribution of leafy vegetables and nuts was relatively low. The statistical analysis revealed a significant positive correlation between TPC and AC. The results of the study indicate the antioxidant potential of the adolescent diet. Conclusions: Despite certain limitations, the values obtained from the study are comparable to those of other studies that employed similar methodologies. Consequently, promoting the early consumption of fresh plant-based foods rich in antioxidants, such as polyphenols, which can enhance the dietary profile and contribute to adolescents’ long-term health, constitutes a significant area of research.

## 1. Introduction

Antioxidants such as polyphenols are bioactive compounds present in a wide variety of plant foods [1]. Various foods, including fruits, vegetables, grains, olive oil, and legumes, have been identified as important sources. Polyphenols are a category of plant secondary metabolites with the potential to modulate inflammatory signaling [2].

In recent years, there has been significant interest in the potential health benefits of polyphenols, primarily their antioxidant, anti-inflammatory, and anticancer effects [3]. Furthermore, several studies have shown that implementing preventive measures during adulthood can effectively mitigate the development of cardiometabolic risk factors, such as obesity, hypertension, and alterations in lipid and glucose metabolism [4]. This assertion is based on the idea that a proper diet during adolescence, a period characterized by profound physiological and social transformations, is of paramount importance [5]. Consequently, a diet based on plant-based foods with a high phenolic content is recommended during this stage due to its ability to maintain optimal cardiometabolic parameters, thanks to the anti-inflammatory and prebiotic properties inherent in these compounds [6]. Additionally, a nutritionally balanced diet, abundant in antioxidants, has been associated with optimal mental well-being [7]. This association is attributed to the role of oxidative stress in the pathophysiology of depression [8].

Antioxidant studies are often conducted individually; however, their effects tend to be more pronounced when foods are integrated (regarding dietary parameters). This is likely due to integrating synergistic dietary patterns [9]. Consequently, the interrelationships between the various compounds in the diet tend to be more pronounced, due to the balance of their ability to support the digestive process and the degree of biological activity at the cellular level [10].

Adolescence represents a critical period of accelerated physical, hormonal, and cognitive development, accompanied by increased vulnerability to metabolic and oxidative stress [11,12]. During this stage, poor dietary patterns, such as high intake of ultra-processed, energy-dense foods and low consumption of fruits and vegetables, can exacerbate oxidative imbalance and contribute to the early onset of chronic non-communicable diseases, including obesity, type 2 diabetes, and hypertension [13,14]. Antioxidants from plant sources, including carotenoids, polyphenols, vitamins, and minerals, play a crucial role in modulating oxidative stress, protecting cell membranes from lipid peroxidation, and supporting immune and metabolic function during growth [15]. Therefore, evaluating antioxidant intake during adolescence is relevant not only from a physiological standpoint but also from a preventive public health perspective, as this stage shapes lifelong dietary behaviors [16].

Some studies have evaluated the antioxidant capacity and bioactive compounds using 24 h recalls and laboratory techniques. Two studies in Spain determined the antioxidant capacity (AC) of plant-based foods and beverages in a Spanish Mediterranean population at different stages of life. Food samples and recipes are also used in the older adult population. The AC of the initial study yielded estimated results of 6014 and 3549 µmol Trolox equivalents/day/person (FRAP and ABTS•^+^), attributing mainly the AC of the diet to beverages, fruits, and vegetables. The second study documented the consumption of a mean value of 332.7 mg/day for polyphenols in the adult population. Data were obtained using five analytical methods: chromatography, chromatography with hydrolysis, Folin assay, differential pH methods, and normal-phase HPLC [17,18]. A study by Wisnuwardani et al., (2019) [14] analyzed an adolescent population aged 12.5 to 17.5 years from eight European countries and reported an intake of 329 mg/day (24 h recall) estimated by the Phenol-Explorer database, chromatography and chromatography after hydrolysis, and for some other foods, polyphenols were calculated based on common brands in the study country. Another study conducted in an adolescent population (56 participants aged 14 to 17 years) in Cosenza, Italy, reported an estimated average daily intake of total polyphenols of 434.46 mg/day (49% flavonoids, 39% phenolic acids, and 12% other polyphenols) [19]. Also, a study was reported in Spanish adolescents aged 11 to 14 years from public schools (Barcelona and Madrid), where the intake of polyphenols was estimated at 598.6 mg/day, evaluated using semi-quantitative food frequency questionnaires and the Phenol-Explore database [20].

On the other hand, in people aged 25 to 65 years in Indonesia, obtaining estimates of consumption of phenolic compounds such as isoflavones (32.20 mg/day), flavonols (15.64 mg/day), flavanones (6.14 mg/day), flavan-3-ols (4.57 mg/day), and flavonoids (3.89 mg/day), in addition to other compounds that were part of the synergy with food consumption [21]. The content was predominantly composed of legumes, vegetables, and fruits. Additionally, the ORAC methodology was used to estimate the AC provided by the diet in young adults in Paraguay. The AC provided in the diet was found at 3093 g/day. The primary sources of these nutrients include fruits, juices, vegetables, cooked vegetables, and legumes [22]. On the other hand, a study to determine the contribution of fruits and vegetables to polyphenol intake and dietary AC in a rural population of Mexican women [23]. The data revealed an average intake of 2442.34 mg/day/person and 1057.45 mg/day/person for FRAP and ABTS•^+^, respectively, and 842.28 mg/day/person for phenol compounds.

Several studies have examined adolescents’ dietary habits, revealing that fruit and vegetable consumption is often insufficient, resulting in a limited intake of bioactive compounds [2,24]. Such is the case of a study on Mexican adolescents [16]. The study used a semi-quantitative food frequency questionnaire for both foods and beverages.

The findings indicated that urban areas consumed more processed meats, botanicals, sweets, and desserts. The southern region of Mexico had the highest fruit consumption, while legumes, eggs, and dairy products were consumed less. The results of this study are consistent with those reported [16]. However, the AC of the diets of Mexican adolescents or adolescents from other countries has not been studied. Consequently, this study offers a more comprehensive perspective, as this developmental period is critical for forming long-term eating habits. Therefore, evaluating bioactive compounds and AC in adolescent diets would provide a more comprehensive perspective, thus facilitating the development of new strategies to promote the consumption of foods with health-beneficial compounds. In this context, the present study sought to quantify Mexican adolescents’ total phenolic compound (TPC) and AC diets.

## 2. Materials and Methods

### 2.1. Study Population and Sampling Strategy

This investigation was founded on the findings of a preceding food frequency questionnaire (FFQ) study [16], which demonstrated reproducibility and consistency through Cronbach’s alpha analysis, yielding a very similar value in both administrations (FFQ1 α = 0.91; FFQ2 α = 0.92). The test–retest reliability was confirmed using the Wilcoxon test, with no significant differences found between the two administrations (Z ≤ 1.96; *p* > 0.05). Therefore, its feasibility for use with adolescents was determined. This descriptive, cross-sectional study employed non-probability convenience sampling, selecting participants based on their availability and willingness to participate. Following the implementation of specific inclusion and exclusion criteria, 178 adolescents (91 females and 87 males) were recruited. The participants, along with their parents or guardians, provided written informed consent. The present study was approved by the Ethics and Research Committee of the Institute of Health Sciences (ICSa) of the Autonomous University of the State of Hidalgo (UAEH), Mexico (Official letter number Cinv/o/0008/2016, code 044). The adolescents between the ages of 14 and 19, enrolled in the public high school of Mineral de la Reforma, Hidalgo, Mexico, were eligible to participate in the study. Participants who were pregnant at the time of data collection were excluded. Food consumption data were collected using a 24 h food frequency questionnaire (FFQ), a validated semi-quantitative instrument for Mexican adolescents [25]. This questionnaire assessed the frequency with which 99 foods from the Mexican Equivalent Foods System (MEFS) were consumed from each food group during the two weeks before the survey date. The questionnaire consisted of three sections. The initial section of the survey included inquiries concerning subject identification and socioeconomic data. The subsequent section enumerated the foods belonging to each MEFS food group. The sequence of food groups is as follows: (1) vegetables, (2) fruits, (3) cereals and tubers, (4) legumes, (5) animal-based foods, (6) milk, (7) oils and fats, and (8) sugars. Finally, the third section included information on the most common food preparations. Trained researchers, food models, and home-cooked portions, teaspoon (5 mL), spoon (15 mL), and cup (240 mL) were used to illustrate adequate amounts according to the MEFS. Daily intake and number of grams of each food were obtained by calculating the total grams from the FFQ and using the MEFS energy reference for each food, since it is mainly used to measure portions or rations of foods, whose nutritional contribution is similar to those of the same group in quality and quantity. The selected food groups were identified based on their frequency of consumption by 15% or more of the adolescent population. The preliminary literature review was conducted on the foods consumed in the study sample, considering those that have demonstrated a sufficient quantity of antioxidants to warrant further investigation.

### 2.2. Sample Selection

The food items included in the study, derived from the previous survey [16], were obtained from local markets and supermarkets in Pachuca, Hidalgo, Mexico, to which the population has access. The collection took place during September and October 2017 to minimize seasonal fluctuations in the antioxidant content of the samples, as the frequency questionnaire had been administered a year earlier during the same months. For each food, three individual units were homogeneously selected for analysis in triplicate. The selection criteria were contingent upon the type of food: (1) Fresh produce (fruits and vegetables): samples should be at their commercial optimal ripeness, without visible bruises, blemishes, or other physical damage; (2) Packaged food products with a valid expiration date and intact packaging. All food samples were transported from the central supply center in Pachuca de Soto, Hidalgo, to the laboratory in the Institute of Health Science in hermetically sealed plastic bags, placed within a dark container to prevent light exposure. Fruits and vegetables were meticulously washed with running water and soap. The outer layer of the produce was gently massaged with the soap, while exercising caution to avoid any damage to the product, and lettuce samples were disinfected with a silver-based sanitizing solution. The specific processing steps were applied to each food group to reflect typical consumption forms. The tomatoes were subjected to thermal treatment (cooking), and juice was extracted before homogenization. In the cereal group, tortillas were lightly toasted, while rice and pasta were rinsed under running water and boiled. In the legumes group, black beans underwent a standardized preparation consisting of rinsing, soaking for 30 min, and cooking in a pressure cooker for one hour. In the fats and oils group, avocados were washed with water and soap to remove surface contaminants. All processed samples were homogenized using a commercial blender, except for lettuce (processed with an immersion blender), rice, and avocado, which were manually ground using a mortar and pestle to achieve uniform particle size.

According to Prayitno et al., (2025) [26] certain foods are processed by removing edible parts, washing and disinfecting, or by cooking in boiling water, frying, roasting, etc., and these processes has the capacity to induce positive or negative modifications, such as a reduction in microbial load, loss of nutritional or bioactive components, alterations in texture, formation of aromas, inactivation of compounds considered antinutritional factors, or even improvement of the bioavailability of some food components, and they should be taken into account in any foods study.

Two main criteria were established to guide the selection of foods for analysis [16]: (1) their inclusion in food groups reported as important sources of antioxidants, such as fruits and vegetables, cereals, legumes, oils and fats, as well as those most consumed by a percentage equal to or greater than 15% within the adolescent population, to evaluate their TPC and antioxidant activity. Among sugars, only chocolate powder was selected. Among the other groups mentioned above, the following foods were selected: cucumber, husk tomato, lettuce, onion, tomato, apple, banana, melon, orange, papaya, bolillo, breakfast cereal, corn tortilla, crackers, flour tortilla, pasta, rice, sugary breakfast cereal, sweet bread, beans, avocado, Japanese peanuts (Mexican snack toasted peanuts topped with a crispy wheat flour coating), natural peanuts, pecan nuts and soy oil.

### 2.3. Phenolic Compound Total and Antioxidant Capacity

#### Extraction of Antioxidants

Phenolic compounds were extracted using a protocol adapted [27], with slight modifications. For each food sample, 250 mg of fresh material was extracted with two solvent systems: methanol: water (50:50, *v*/*v*) and acetone: water (70:30, *v*/*v*), both applied at a ratio of 40 mL per gram of sample. The extraction process involved constant agitation at ambient temperature (approximately 25 °C) for 30 min with each solvent. The solutions were centrifuged (EE.UU., Beckman Coulter Allegra™ 25R, Brea, CA, USA) at 3000 rpm for 30 min. Supernatants from both solvent systems were combined and stored at 4 °C in amber vials.

### 2.4. Total Phenolic Compounds

TPC quantification was performed using the Folin–Ciocalteu colorimetric method [28], with adaptations for microplate analysis. One hundred µL of the sample extract was combined with 500 µL of Folin–Ciocalteu reagent, diluted 1:10. This mixture was then followed by adding 400 µL of 7.5% sodium carbonate. The reaction mixture was subjected to an incubation process at ambient temperature for 30 min to safeguard it from exposure to light. The absorbance was measured at 765 nm using a microplate spectrophotometer (EE. UU., PowerWave XS UV, BioTek Instruments, Winooski, VT, USA) and analyzed with KC Junior software v1.41.8. TPC values were calculated from a calibration curve (R^2^ = 0.999) prepared with gallic acid standards and expressed as milligrams of gallic acid equivalents per gram of fresh weight (mg GAE/g fw).

### 2.5. Antioxidant Capacity

ABTS•^+^ and DPPH• were used, as DPPH• has been documented to be more applicable to phenolic compounds [29]. However, whenever possible, it is recommended to use the ABTS•^+^ assay in conjunction with DPPH• to determine the antiradical activity of the analyzed samples. The radicals used are not chemically identical, which facilitates contact with some antioxidants dissociated into anions [30].

The AC of food samples was evaluated by the Folin–Ciocalteu, ABTS•^+^, and DPPH• assays. These methods were selected for their analytical accuracy and sensitivity in evaluating phenolic compounds, as well as their ability to absorb radicals. The Folin–Ciocalteu method primarily estimates total phenolic content via redox reactions, whereas the ABTS•^+^ and DPPH• tests assess the ability of antioxidants to quench free radicals via electron- or hydrogen-atom-transfer mechanisms.

However, these in vitro trials have limitations. They do not account for key physiological variables such as compound bioavailability, interactions within the food matrix, or metabolic changes after ingestion. Therefore, the antioxidant values obtained should be seen as indicating possible antioxidant activity under controlled conditions, rather than as direct evidence of effectiveness in vivo. These limitations are inherent to chemical testing and highlight the need for complementary approaches in future research.

The assessment of AC was conducted through the implementation of two colorimetric tests. ABTS•^+^ and DPPH• (2,2-diphenyl-1-picrylhydrazyl) were analyzed according to the procedures outlined [31,32], with minor modifications. The radical cation ABTS•^+^ was generated by combining a 7 mmol/L solution with 2.45 mmol/L potassium persulfate, followed by dark incubation at room temperature (approximately 25 °C) for 16 h. The resulting radical solution was then diluted with ethanol to absorb 0.70 ± 0.10 at 754 nm. The sample extract (20 μL) was mixed with a diluted ABTS•^+^ solution (980 μL) and subsequently incubated for seven minutes at room temperature. A calibration curve was prepared (R^2^ = 0.996), with ascorbic acid standard. The resulting change in the color of the samples was measured at a wavelength of 754 nm. The AC was expressed in milligrams of ascorbic acid equivalents per gram of fresh weight (mg EAA/g fw). The DPPH• assay was performed using an ethanolic solution of DPPH•, with a concentration of 7.4 milligrams per 100 milliliters. For each test, 100 μL of the sample extract was mixed with 500 μL of DPPH• solution and allowed to react for 1 h at room temperature. A calibration curve was prepared (R^2^ = 0.998), with Trolox standard. The absorbance was measured at a wavelength of 520 nm. The results were reported as Trolox micromoles equivalent per gram of fresh weight (μmol TE/g fw).

### 2.6. Estimation of Dietary Antioxidant Intake

Antioxidant intake and AC were estimated using established equations, these assays do not account for bioavailability, the effects of each food, or in vivo activity; however, they are based on quantified concentrations of phenolic compounds and antioxidant activity, as well as grams obtained from the consumption frequency data reported in the adolescent food frequency questionnaire [16].

These calculations indicate daily intake levels in adolescents as follows:

Intake of TPC = (g) × (PC in each food)

Intake of foods with AC = (g) × (AC in each food)

Where

TPC = Total Polyphenolic Content

PC = Polyphenolic Content

AC = Antioxidant Capacity

g = grams of fresh matter of edible portion/day/person

### 2.7. Statistical Analysis

Descriptive statistics (mean and standard deviation) were calculated for each food item concerning the total phenolic content (TPC) and antioxidant capacity (AC).

One-way analysis of variance (ANOVA), followed by Tukey’s post hoc test, was used to compare TPC and AC among different food groups. Data were presented as mean ± standard deviation (SD), and the statistical significance was defined as *p* < 0.05. The association between the dependent variables (CPT, ABTS•^+^, DPPH•) was assessed using Spearman’s correlation

All statistical analyses were conducted with SPSS version 24 (IBM Corp., Armonk, NY, USA).

## 3. Results

### 3.1. Total Phenolic Content in Foods and Estimated Intake Among Mexican Adolescents

The foods selected for this analysis were obtained from the research conducted by Escamilla-Gutiérrez et al. (2025) [16]. A total of 26 foods were selected for inclusion in the study. Five vegetables, five fruits, nine cereals, one legume, five oils and fats, and one sugar, identified as powdered chocolate, were identified within the food groups.

All analyzed foods exhibited substantial variability in their TPC concentrations. Among vegetables and fruits, TPC values ranged from 0.16 to 2.29 mg GAE/g and 1.20 to 2.05 mg GAE/g, respectively (Table 1). Onion, papaya, and banana were identified as the richest sources of phenolic compounds within their categories, with significant statistical differences from other items in their respective groups (*p* < 0.05). In the cereal group, TPC concentrations ranged from 0.06 to 1.80 mg GAE/g, with the highest values observed in saltine crackers, sweet breads, buns, and breakfast cereals. Legumes, particularly beans, exhibited a TPC of 2.02 mg GAE/g. Within the fats and oils category, pecan nuts showed the highest overall TPC value (21.87 mg GAE/g), significantly exceeding that of all other food groups (*p* < 0.05).

The AC of the food samples was evaluated through the use of two different assays: ABTS•^+^ and DPPH•. These assays yielded complementary antioxidant activity profiles. As illustrated in Table 1, the AC values obtained through the ABTS•^+^ assay varied from 0.03 to 4.96 mg EAA/g, while those obtained via the DPPH• assay ranged from 0.78 to 63.13 µmol TE/g (Table 1). In both assays, Onion exhibited the highest AC among vegetables, with statistically significant differences (*p* < 0.05). Among the fruits examined, melon and banana showed values in the ABTS•^+^ assay, while papaya demonstrated a notable response in the DPPH• assay. In the cereal group, the pasta exhibited the highest AC, as determined by the ABTS•^+^ assay (2.16 mg AAE/g). At the same time, white bread and corn tortillas demonstrated the highest values in the DPPH• assay (173.96 and 1131.23 µmol TE/day, respectively) (Table 1). Among processed foods, powdered chocolate ranked third in AC according to ABTS•^+^ (1.28 mg AAE/g) and second overall in DPPH• (38.04 µmol TE/g), among the top three food sources in both assays. In the fats and oils group, pecan nuts obtained the highest AC (*p* < 0.05), measuring 4.96 mg AAE/g (ABTS•^+^) and 148.40 µmol TE/g (DPPH•), followed by Japanese-style and natural peanuts. Despite their high antioxidant content, these foods were not necessarily the most significant contributors to dietary AC among adolescents. Conversely, the foods consumed in larger quantities functioned as the predominant dietary sources of antioxidants. The estimated dietary antioxidant intake was 345.67 mg AAE/person/day, based on the ABTS•^+^ assay, and 5399.14 µmol TE/person/day, in the DPPH• assay.

### 3.2. Antioxidant Capacity of Foods and Estimated Dietary Intake Among Adolescents

Based on these concentrations and reported consumption patterns, the estimated daily intake of phenolic compounds among adolescents was 1484.01 mg GAE/person/day (Table 2). Within food groups, onion, apple, banana, papaya, corn tortillas, beans, and avocado were the main contributors to phenolic intake due to their high compound concentration or frequent consumption.

According to Table 1 and Table 2, the cereals constituted the predominant source of antioxidants, contributing 57 and 59% of the total antioxidant capacity as measured by ABTS•^+^ and DPPH•, respectively. Subsequently, fruits and vegetables (27 and 29%), legumes (8 and 5%), and fats and oils (8 and 7%). In the context of the capacity by ABTS•^+^, onions, melons, bananas, pasta, and pecan nuts were identified as the primary contributors to dietary AC. In contrast, the DPPH• assay revealed that tomatoes with peel, apples, corn tortillas, beans, and pecan nuts were the primary contributors.

In addition, Spearman correlation analysis revealed a significant positive association between TPC and AC, as measured by ABTS•^+^ (r = 0.57, *p* < 0.01) and DPPH• (r = 0.50, *p* < 0.01), supporting a functional relationship between the two variables.

## 4. Discussion

Understanding the antioxidant quality of the diet during adolescence is critical because increased nutritional needs, oxidative stress, and the formation of long-term eating habits characterize this stage of life [33,34]. The present study quantified the intake of TPC and AC from foods commonly consumed by Mexican adolescents, providing new insights into the bioactive potential of their diet. Phenolic compounds are found in various foods and beverages, predominantly of plant origin, as they are secondary metabolites in these tissues. These are naturally present in vegetables and fruits [35,36,37].

Fruits and vegetables are acknowledged as a significant source of phenolic compounds [38,39], as demonstrated by the findings of this study. A comparison of the items, including but not limited to apples, bananas, papayas, melons, onions, and lettuce, exhibited TPC values comparable to those reported in other studies [20,40,41]. Similarly, the phenolic content in avocados, natural peanuts, and pecans is consistent with the findings of studies that have underscored the antioxidant potential of nuts and oilseeds [42].

The intake of TPC in the present study to the adolescent population (~1484 mg GAE/person/day) was higher than the average reported in Mexican women (~842.28 mg/day) [23] or as others studies carried on in older adults on the island of Mallorca (~332.7 mg/day) [18] on people aged 25 to 65 in Indonesia (~62.44 mg/day) [21]. The polyphenol intake was even higher than that assessed in other studies of European children (~329 mg/day) [43], Spanish (~598.6 mg/day) [44], and Italian children and adolescents (~434.46 mg/day) [19]. Some studies on the Mediterranean diet have indicated that the highest levels of phenolic compounds are attained through consuming beverages such as coffee, tea, and wine. However, in the present study, no beverages were evaluated, as sugary fruit drinks and coffee consumption are high among the Mexican adolescent population [45,46,47].

The promotion of the consumption of polyphenol-rich foods from an early age should be regarded as a public health priority, not only for their antioxidant potential but also for their broader role in the prevention of chronic diseases and the improvement of long-term nutritional quality [48]. The hypothesis that dietary patterns characterized by the consumption of fruits, vegetables, legumes, and minimally processed plant-based products can significantly enhance the antioxidant quality of adolescent diets is supported by the presence of bioactive compounds in commonly consumed foods [49]. Therefore, this study is relevant not only for considering food consumption in general, but also for considering the antioxidant content of these foods. This could lead to the development of a more complete diet for adolescents, which would benefit their long-term health by preventing various diseases in adulthood. Furthermore, quantifying antioxidants in the diet could generate important health markers for consumers.

According to the AC analysis, it was found that processed foods, such as bread, breakfast cereals, and powdered chocolate, exhibited notable antioxidant capacity, particularly in the DPPH• assay. This may be due to the formation of Maillard reaction products during thermal processing, which has been described as a mechanism contributing to antioxidant potential in industrially processed products [50,51]. The antioxidant values of chocolate powder, particularly, were higher than those of several fruits and aligned with data reported by other studies [52,53]. This could be due to the AC of cocoa, as attributed to its phenolic and procyanidin content [54].

Despite the absence of an official standard that dates the Recommended Daily Intake (RDI) standardized for total antioxidant capacity, epidemiological studies could be considered that estimated that a total antioxidant capacity of 4.6 µM TE per calorie consumed in the diet should be achieved, which represents 9200 µM TE for a 2000-calorie diet [55,56]. Therefore, a significant positive correlation was observed between TPC and AC for both ABTS•^+^ (r = 0.57; *p* < 0.01) and DPPH• (r = 0.50; *p* < 0.01), confirming the essential role of phenolic compounds that contribute to AC. However, some foods with high TPC displayed moderate AC, suggesting the presence of other bioactive compounds or differences in the chemical nature and reactivity of individual phenolics, as well as matrix effects or synergistic interactions.

Although several foods displayed high antioxidant potential, their dietary contribution was determined by their compound concentration and frequency of consumption. For example, onions, melons, bananas, pasta, and pecan nuts were top contributors to AC as measured by ABTS•^+^, This AC could be due to the antioxidants compounds present in these foods such as flavonoids, quercetin, kaempferol, anthocyanidin stilbene, benzoic acid and derivatives, hidroxycinnamic acid, apigenin, caffeic acid, chlorogenic acid, naringenin, rosmarinic acid, phenolic acids and caffeic acid phenethyl ester [41,44,57,58,59,60]. In contrast, husk tomato, apples, corn tortillas, beans, and pecan nuts ranked highest, phenolic acids and flavonoids [43,44,61,62,63]. Overall, cereals accounted for more than 50% of the daily antioxidant intake in this adolescent population, with estimated values of 345.67 mg AAE/person/day (ABTS•^+^) and 5399.14 µmol TE/person/day (DPPH•). These results are lower than those obtained by Saura-Calixto and Goñi (2006) [17] in a Mediterranean Spanish population and comparable to those reported in Mexican adult women [23], although the sources of antioxidants differ markedly or are reduced in quantity from food groups compared to those in this study.

A study involved a sample of university students in Paraguay [22]. This study’s antioxidant capacity was predominantly derived from sources such as fruits and vegetables (raw and cooked).

The results confirm that foods frequently consumed by adolescents in Mexico contain varying but significant concentrations of phenolic compounds and demonstrate measurable AC. These bioactive properties are influenced by the type of food, its ripeness, processing methods, and frequency of consumption, factors that account for the heterogeneity of antioxidant composition [64,65]. Despite the low variability in the consumption of foods rich in bioactive compounds, adolescents demonstrated similarities in the consumption of antioxidants and CA compared to the studies that were reviewed.

From an evidence-based medicine perspective, this research provides descriptive observational data rather than data to test hypotheses or conduct interventions. A cross-sectional design, a convenience sample, intake estimates based on the FFQs, and in vitro antioxidant assays provide evidence for causal inference. However, the results give specific background information on dietary exposure to phenolic compounds and AC in adolescents, a group often underrepresented in antioxidant research. This type of evidence can serve as a foundation for designing future longitudinal or intervention studies to verify specific hypotheses about antioxidant intake and its effects on health.

### Study Limitations

This study is relevant for considering food consumption of adolescent diets measured by clinical tools, such as recall questionnaires, and antioxidant components in food as eaten in in vitro studies. This could lead to the development of recommendations for a more complete and standardized diet, which would benefit their long-term health by preventing various diseases in adulthood and improving their performance during adolescence. However, it is important to consider some methodological aspects.

Although widely accepted and validated for dietary assessment, semi-quantitative food frequency questionnaires (FFQs) are prone to recall bias and misclassification. Nevertheless, this tool allowed for an efficient estimation of consumption patterns in a sample of adolescents.

Beverages such as tea, coffee, and fruit juices were not included in the intake estimate, which could lead to an underestimation of total antioxidant intake. However, focusing on solid foods allowed for a more specific assessment of antioxidant density in the adolescent food environment.

On the other hand, the sampling was conducted during a specific season and period, reflecting actual consumption within a school year, but this may limit its applicability to other contexts or seasons, since seasonal variations in food are a natural phenomenon throughout the year, influenced by changes in environmental conditions, including weather and pollution levels. Therefore, seasonality is a difficult factor to control. However, the location of origin does not experience the extreme temperature fluctuations seen in other areas, so the measurements were taken during the same months as the questionnaire. This ensures greater control over the variability of antioxidant content. Furthermore, given the potential influence of various factors on the consumption or absence of antioxidants, as well as their quantity, a food consumption questionnaire was administered to establish a baseline for the consumption of specific foods, which could contribute to estimating intake.

It is also important to recognize that the cross-sectional design restricts causal inference; however, it offers a valuable snapshot of antioxidant dietary patterns during a critical developmental stage, paving the way for future longitudinal studies. These variables will not be considered as dependent; however, future studies could be enriched by taking each of these factors into account, emphasizing that the primary contributions of the research lie in delineating dietary baseline exposure to phenolic compounds and antioxidant capacity within adolescent populations.

Ideally, the antioxidant effects of foods should be consistent with in vivo studies (blood, feces, organs), considering all nutritional, physiological, and ecological factors that influence nutrient absorption. However, to study physiological benefits, in vitro methods offer an attractive alternative to human and animal studies, providing complementary information on reducing power and free radical scavenging activity. They can be simple, rapid, and inexpensive, and although assays, such as Folin–Ciocalteu, DPPH•, and ABTS•^+^, do not consider bioavailability or metabolic transformations, they remain a widely used and standardized method for estimating antioxidant potential in food matrices. The Folin–Ciocalteu assay estimates total phenolic content by measuring the reduction in a phosphomolybdic-phosphotungstic complex by phenolic and other reducing compounds in the sample. The ABTS•^+^ assay measures radical scavenging capacity, as antioxidants in the samples reduce the preformed ABTS•^+^ radical cation. The DPPH• assay is based on the ability of antioxidants to donate electrons or hydrogen atoms to the stable DPPH• radical. However, future research could complement these findings with in vivo assessments or bioaccessibility models.

## 5. Conclusions

Adolescence represents a critical period for the establishment of dietary habits that may significantly influence long-term health outcomes. The present study demonstrated that foods frequently consumed by Mexican adolescents contain significant phenolic compounds and exhibit measurable AC, analogous to values reported for other studies. The average intake of TPC was approximately 1484 mg GAE/person/day, with fruits, cereals, and legumes as the main contributors. Regarding AC, adolescents showed estimated intakes of 345.67 mg AAE/person/day (ABTS•^+^) and 5399.14 µmol TE/person/day (DPPH•), indicating a functionally acceptable consumption pattern, albeit with potential for enhancement. Foods like onions, papayas, bananas, beans, bread, tortillas, pasta, and pecans were identified as key sources of bioactive compounds. However, the limited contribution of certain food groups, such as green leafy vegetables and nuts, underscores the need to encourage their inclusion in the daily diet. These findings indicate a lack of diversity in adolescents’ diets. Despite this observation, the daily intake of phenolic compounds and antioxidant capacity obtained in this study are similar to the estimated antioxidant consumption reported in other studies conducted in different populations and for the Mediterranean diet on different populations. However, there is a need to promote dietary patterns based primarily on fresh, minimally processed, culturally appropriate, and accessible fruits and vegetables to improve the antioxidant profile of the diet from the earliest stages of life.

## Figures and Tables

**Table 1:** Total phenolic compounds and antioxidant capacity by ABTS•^+^ and DPPH• in different food groups per gram of sample.

Foods	Total Phenolic Compounds mg GAE/g fw	Antioxidant Capacity	
		ABTS•^+^ mg AAE/g fw	DPPH• μmol TE/g fw
Vegetables			
Cucumber	1.15 ± 0.10 ^D^	0.16 ± 0.01 ^A^	1.39 ± 0.13 ^A^
Husk Tomato	0.92 ± 0.08 ^C^	0.09 ± 0.01 ^B^	1.75 ± 0.15 ^C^
Lettuce	0.78 ± 0.04 ^B^	0.23 ± 0.02 ^B^	2.58 ± 0.23 ^B^
Onion	2.29 ± 0.21 ^E^	0.58 ± 0.02 ^B^	2.78 ± 0.17 ^D^
Tomato	0.16 ± 0.01 ^A^	0.11 ± 0.01 ^B^	1.48 ± 0.15 ^A^
Fruits			
Apple	1.20 ± 0.12 ^A^	0.07 ± 0.01 ^A^	3.30 ± 0.25 ^C^
Banana	1.90 ± 0.13 ^D^	0.12 ± 0.01 ^B^	1.28 ± 0.12 ^A^
Melon	1.62 ± 0.14 ^C^	0.13 ± 0.01 ^B^	2.75 ± 0.26 ^B^
Orange	1.44 ± 0.06 ^B^	0.10 ± 0.01 ^B^	2.63 ± 0.24 ^B^
Papaya	2.05 ± 0.10 ^E^	0.11 ± 0.01 ^B^	3.70 ± 0.35 ^D^
Cereals			
Bolillo	1.79 ± 0.11 ^E^	0.11 ± 0.01 ^AB^	27.85 ± 1.59 ^F^
Breakfast Cereal	1.70 ± 0.11 ^E^	0.11 ± 0.01 ^AB^	4.15 ± 0.06 ^D^
Corn Tortilla	1.53 ± 0.13 ^D^	0.14 ± 0.01 ^AB^	27.11 ± 1.60 ^F^
Crackers	1.80 ± 0.05 ^E^	0.24 ± 0.02 ^C^	1.47 ± 0.15 ^AB^
Flour Tortilla	0.98 ± 0.07 ^B^	0.09 ± 0.01 ^A^	0.78 ± 0.09 ^A^
Pasta	0.06 ± 0.00 ^A^	2.16 ± 0.14 ^D^	1.42 ± 0.14 ^AB^
Rice	0.15 ± 0.01 ^A^	0.08 ± 0.01 ^A^	24.79 ± 0.99 ^E^
Sugary Breakfast Cereal	1.31 ± 0.10 ^C^	0.36 ± 0.03 ^D^	2.48 ± 0.19 ^BC^
Sweet Bread	1.72 ± 0.10 ^E^	0.17 ± 0.02 ^BC^	3.53 ± 0.21 ^CD^
Legumes			
Beans	2.02 ± 0.10	0.36 ± 0.02	3.50 ± 0.33
Oils and fats			
Avocado	4.75 ± 0.38 ^BC^	0.21 ± 0.02	3.19 ± 0.22 ^AB^
Japanese Peanuts	4.34 ± 0.19 ^BC^	0.59 ± 0.05	6.89 ± 0.67 ^C^
Natural Peanuts	3.86 ± 0.31 ^B^	0.42 ± 0.02	1.76 ± 0.12 ^BC^
Pecan Nuts	21.87 ± 0.94 ^D^	4.96 ± 0.49	63.13 ± 2.33 ^D^
Soy Oil	0.10 ± 0.01 ^A^	0.03 ± 0.00	0.82 ± 0.01 ^A^
Sugars			
Powdered Chocolate	5.13 ± 0.30	1.28 ± 0.09	38.04 ± 3.14

Values are presented as mean ± standard deviation. ^A–F^ denotes statistically significant differences among each food group (*p* < 0.05).

**Table 2:** Contribution of polyphenol compounds and antioxidant capacity to the adolescent diet.

Foods	g Fresh Matter of Edible Portion/Day/Person *	^1^ Total Phenolic Compounds	Antioxidant Capacity	
			^2^ ABTS•^+^	^3^ DPPH•
Vegetables				
Cucumber	62.52 ± 94.01	71.90 ± 6.25	9.73 ± 0.87 ^D^	86.70 ± 7.98 ^B^
Husk Tomato	75.36 ± 122.04	69.40 ± 6.42	6.46 ± 0.73 ^B^	131.88 ± 11.69 ^E^
Lettuce	35.12 ± 47.18	27.39 ± 1.40	8.31 ± 0.72 ^C^	90.61 ± 8.25 ^C^
Onion	40.28 ± 66.37	92.28 ± 8.49 *	23.51 ± 0.96 ^A^	112.04 ± 6.88 ^D^
Tomato	53.65 ± 84.01	8.70 ± 0.96	5.72 ± 0.44 ^E^	79.58 ± 8.21 ^A^
Fruits				
Apple	118.75 ± 163.66	142.97 ± 14.38 *	8.05 ± 0.79 ^C^	392.70 ± 29.80 ^E^
Banana	74.09 ± 114.75	140.60 ± 10.06 *	9.11 ± 0.99 ^D^	94.71 ± 8.94 ^A^
Melon	75.26 ±126.18	122.14 ± 10.62	9.62 ± 1.05 ^E^	211.05 ± 23.28 ^C^
Orange	46.89 ± 76.59	67.76 ± 3.02	4.53 ± 0.33 ^A^	123.28 ± 11.13 ^B^
Papaya	67.60 ± 124.80	138.52 ± 7.06 *	7.51 ± 0.63 ^B^	249.82 ± 23.67 ^D^
Cereals				
Bolillo	27.71 ± 41.24	49.58 ± 3.02	3.05 ± 0.26 ^D^	771.84 ± 44.14 ^F^
Breakfast Cereal	5.11 ± 9.64	8.71 ± 0.55	0.56 ± 0.04 ^A^	21.21 ± 0.30 ^C^
Corn Tortilla	41.73 ± 44.86	63.85 ± 5.34 *	5.95 ± 0.49 ^F^	1131.23 ± 66.84 ^H^
Crackers	5.29 ± 10.48	9.53 ± 0.28	1.26 ± 0.12 ^B^	7.81 ± 0.77 ^A^
Flour Tortilla	21.01 ± 35.36	20.51 ± 1.43	1.90 ± 0.19 ^C^	16.35 ± 1.85 ^B^
Pasta	80.60 ± 105.81	5.07 ± 0.61	173.96 ± 11.41 ^G^	114.45 ± 11.07 ^D^
Rice	38.64 ± 48.79	5.75 ± 0.56	3.17 ± 0.32 ^A^	957.98 ± 38.10 ^G^
Sugary Breakfast Cereal	8.48 ± 11.67	11.09 ± 0.90	3.05 ± 0.28 ^D^	21.10 ± 1.64 ^C^
Sweet Bread	34.60 ± 48.20	59.36 ± 3.70 *	5.92 ± 0.57 ^F^	122.21 ± 7.23 ^E^
Legumes				
Beans	73.17 ± 96.79	147.44 ± 6.58	26.34 ± 1.30	256.09 ± 23.93
Oils and fats				
Avocado	19.26± 32.00	91.48 ± 7.39 *	4.12 ± 0.39 ^C^	61.48 ± 4.35 ^D^
Japanese Peanuts	7.43 ± 15.09	32.23 ± 1.41	4.39 ± 0.41 ^C^	51.18 ± 4.97 ^C^
Natural Peanuts	7.43 ± 15.09	28.70 ± 2.30	3.15 ± 0.16 ^B^	13.05 ± 0.91 ^A^
Pecan Nuts	2.35 ± 4.57	51.39 ± 4.57	11.65 ± 1.16 ^D^	148.40 ± 5.48 ^E^
Soy Oil	27.78	2.90 ± 0.31	0.97 ± 0.15 ^A^	22.85 ± 0.39 ^B^
Sugars				
Powdered Chocolate	2.88 ± 4.78	14.76 ± 0.88	3.68 ± 0.27	109.54 ± 9.04
Total daily intake		1484.01	345.67	5399.14

* These data were obtained from Escamilla-Gutiérrez et al. (2025) [16], which are considered foods with a higher content of TPC among the same group. ^1^ mg GAE/day/person; ^2^ mg AAE/day/person; ^3^ μmol TE/day/person; ^A–H^ indicate significant differences among the same group of food (*p* < 0.05).

## Data Availability

The original contributions presented in this study are included in the article. Further inquiries can be directed to the corresponding authors.
